# Raynaud's phenomenon during treatment with lisdexamfetamine: risk of cerebral vasospasm?

**DOI:** 10.31744/einstein_journal/2025RC1439

**Published:** 2025-10-03

**Authors:** Rubens Pitliuk, Tatyanny Paula Pinto da Costa Santos Fucci

**Affiliations:** 1 Hospital Israelita Albert Einstein São Paulo SP Brazil Hospital Israelita Albert Einstein, São Paulo, SP, Brazil.; 2 Nutricionist São Paulo SP Brazil Nutricionist, São Paulo, SP, Brazil.

**Keywords:** Attention deficit disorder with hyperactivity, Raynaud disease, Lisdexamfetamine, Drug therapy, Attention deficit disorders, Dizziness, Vasospasm, intracranial

## Abstract

Lisdexamfetamine, a prodrug used to treat Attention Deficit/Hyperactivity Disorder in children, adolescents, and adults, is an inactive substance that is converted into its active form (dextroamphetamine) after being metabolized. This conversion primarily occurs in the bloodstream through enzymatic cleavage following active absorption from the gastrointestinal lumen. The active metabolite then stimulates the central nervous system by increasing the levels of dopamine and norepinephrine in the brain. It was discovered in 1996 by New River Pharmaceuticals and approved by the FDA in 2007 for the treatment of Attention Deficit/Hyperactivity Disorder in children. In this article, two cases of secondary Raynaud's phenomenon due to the use of lisdexamfetamine are described. Raynaud's disease is the primary form, occurring in the absence of an underlying cause, and differs from secondary Raynaud's phenomenon, which is associated with various medical conditions and pharmacological agents. In the cases reported in this study, the phenomenon occurred during treatment with lisdexamfetamine, where it is listed as an uncommon adverse effect in the prescribing information. In both patients presented in this report, discontinuation of the medication led to resolution of the phenomenon within a few days. This report highlights the fact that one of the patients reported episodes of dizziness during Raynaud's phenomenon, drawing attention to the potential associated complications.

## INTRODUCTION

Raynaud's phenomenon (RP) is a relatively common clinical syndrome characterized by distinctive color changes in the fingers resulting from vasospasms. These changes can be triggered by cold environments, emotional stress, or other physical or medicinal exposures.^(1)^

Most individuals with RP have primary Raynaud's condition; however, Raynaud's disease can also occur secondarily to a wide range of medical conditions and treatments. Unlike primary Raynaud's disease, patients with secondary Raynaud's disease may develop persistent digital ischemia, including ulcers and gangrene. These patients require thorough clinical evaluation and investigation, particularly for the detection of autoantibodies and nailfold capillaroscopic abnormalities.^(2)^

This report presents two patients who experienced RP during lisdexamfetamine dimesylate (LDX) treatment.

Lisdexamfetamine dimesylate is a long-acting prodrug used to treat attention-deficit/hyperactivity disorder (ADHD) in children, adolescents, and adults.^(3)^ The therapeutic benefits of LDX are achieved within 1.5 hours of administration and can last up to 13 hours, with efficacy comparable or superior to that of other available psychostimulants. The literature also documents long-term efficacy with safety and tolerability profiles comparable to those of other stimulants used for the treatment of ADHD. Most adverse events associated with LDX are mild or moderate in severity, with appetite loss and insomnia being the most common.^(4)^

A previous study reported that, although the association between RP and LDX is supported by a limited number of cases, the World Health Organization and Eudravigilance databases suggest a possible causal relationship between RP and LDX.^(5)^

In clinical practice, it is crucial to differentiate between patients with primary and secondary RP and to predict which patients with RP will progress to another disease, particularly autoimmune rheumatic diseases. This differentiation is extremely important for determining the severity, prognosis, and most appropriate treatment.^(6)^

In addition, approximately 10% of patients initially diagnosed with primary RP subsequently develop an autoimmune rheumatic disease, highlighting the importance of identifying patients with a significant risk of developing these conditions.^(6)^

## CASE REPORT

**Figure 1:**
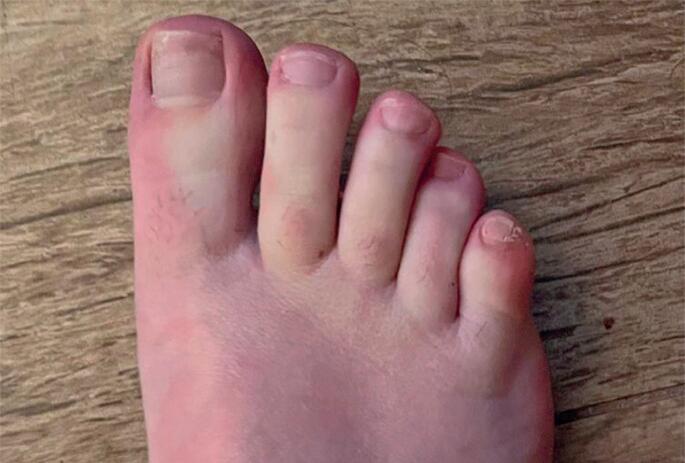
Paleness of the toes during an episode of RP due to decreased blood flow

Patient 1: A 28-year-old female diagnosed with ADHD was first prescribed LDX in August 2023. Her previous treatments included methylphenidate and methylphenidate OROS, to which her ADHD was not responsive. By September 2023, she was receiving 45mg/day of LDX, prepared as divided capsules at a compounding pharmacy. At this time, the patient developed RP, which appeared symmetrically on her hands and feet (Figure 1), and intense dizziness. Due to the significant improvement in her ADHD symptoms with LDX, the patient independently discontinued and later restarted the medication several times, with each reintroduction leading to recurrence of RP and severe dizziness. Episodes of intense dizziness were characterized by a sudden dimming of vision and rotational vertigo and lasted a few minutes. These episodes occurred without exposure to abrupt movements or sudden changes in position. The LDX was discontinued, leading to no further episodes of RP or dizziness.

**Figure 2:**
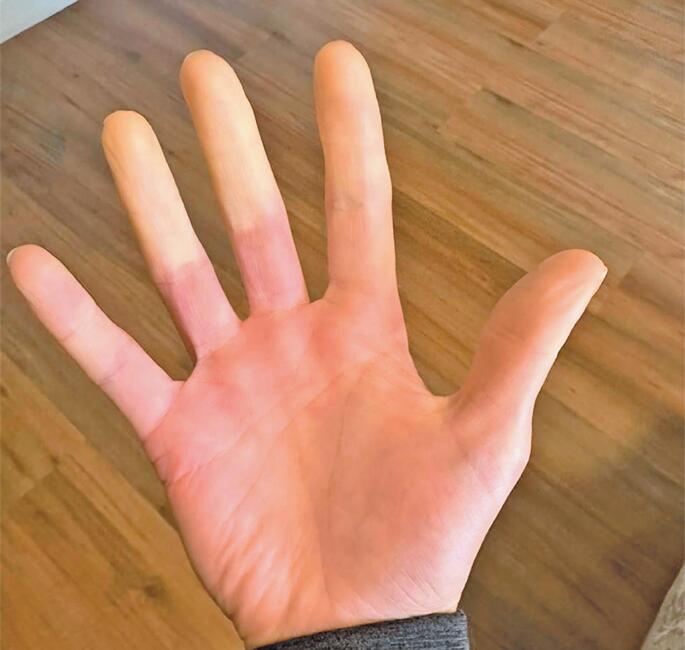
Evident pallor in the upper third of the fingers during the RP episode. The pallor is due to decreased blood flow

Patient 2: A 43-year-old woman diagnosed with ADHD and type II bipolar disorder (well-stabilized with lithium carbonate and escitalopram) was first prescribed LDX in 2022. She had previously been prescribed methylphenidate (30mg/day) for 8 years with unsatisfactory improvement of her ADHD. At an LDX dose of 70mg/day, the patient reported complete improvement of her ADHD symptoms without the "peaks and valleys" she felt with methylphenidate. In May 2023, the patient presented with RP in her hands (Figure 2). She discontinued LDX and her symptoms disappeared within a few days. The patient reported previously experiencing RP, but only on very cold days. She sought emergency care at *Hospital Albert Einstein* in August 2023, at which time reversible cyanosis that was not associated with very cold temperatures, but related to an increased dose of Venvanse, was noted in her hands and feet. She was referred to a rheumatologist and advised to taper her Venvanse dose as she did not exhibit symptoms suggestive of other conditions such as collagenosis.

The Ethics and Research Committee of *Hospital Israelita Albert Einstein* approved this study (CAAE: 83159224.2.0000.0071; # 7.092.650). The information and photographs presented in this article were obtained with patient consent.

## DISCUSSION

This report highlights the importance of detailed patient evaluation and individualized considerations. Patient 1 experienced intense dizziness and physical signs of RP; however, cerebral symptoms were not observed in Patient 2.

Based on these observations, it remains unclear whether dizziness is an isolated symptom or related to RP. The impact of this symptom on the body warrants further attention, and the potential systemic consequences of cerebrovascular complications require further investigation.

Vasospasms associated with RP secondary to LDX use may extend beyond peripheral circulation to affect the central nervous system, potentially resulting in severe neurological symptoms. Vasospasms reduce the lumen of blood vessels, consequently decreasing blood flow and oxygen and nutrient supply to brain tissue, which may result in focal or diffuse ischemia. Clinically, these changes can manifest as headaches, dizziness, motor and sensory deficits, or seizures.

Furthermore, recurrent vasospasms can compromise the integrity of the blood-brain barrier, leading to fluid leakage and vasogenic edema. Raynaud's phenomenon, associated with psychostimulant use, is primarily attributed to the peripheral release of catecholamines, which promote vasoconstriction. Methylphenidate, another psychostimulant widely used for the treatment of ADHD, also acts as a catecholamine reuptake inhibitor and may induce RP.

A previous case report described the worsening of symptoms in a young patient with a history of Raynaud's episodes after the use of Adderall, which had been prescribed for the treatment of oppositional defiant disorder and ADHD. Following a careful review of the patient's medical history, Adderall was discontinued and an improvement in symptoms was reported.^(7)^

Therefore, LDX should be used with caution in patients who are at risk of developing RP. Furthermore, a thorough investigation and detailed understanding of these cases, particularly in the context of medication use, are crucial to prevent greater harm to patients. Among these potential harms is the discomfort caused by this adverse reaction, described by patients as "distressing episodes."^(8)^

## CONCLUSION

Although lisdexamfetamine dimesylate is widely used to treat attention-deficit/hyperactivity disorder, its potential to induce Raynaud's phenomenon requires careful consideration. This rare adverse effect highlights the importance of vigilant monitoring for vasospastic episodes.

This report is limited by the absence of neuroimaging examinations, which could have clarified the presence of structural changes such as vasogenic edema or signs of ischemia. Future studies should include such imaging and investigate the underlying mechanisms of psychostimulant-induced Raynaud's phenomenon in detail to better elucidate its pathophysiology and clinical implications.

Finally, for patients who continue lisdexamfetamine dimesylate despite the development of Raynaud's phenomenon, a comprehensive evaluation of potential long-term risks is essential. This is particularly important for individuals predisposed to vascular disorders or other underlying rheumatological conditions.
